# Effect of ulinastatin on growth inhibition, apoptosis of breast carcinoma cells is related to a decrease in signal conduction of JNk-2 and NF-κB

**DOI:** 10.1186/1756-9966-31-2

**Published:** 2012-01-05

**Authors:** Hong Wang, Xin Sun, Feng Gao, Biao Zhong, Yong-hua Zhang, Zhijun Sun

**Affiliations:** 1 Surgery Department of Breast and Thyroid, Second Affiliated Hospital of Chongqing Medical University, Chongqing 400010, China

**Keywords:** Ulinastatin, Taxotere, Breast cancer, Proliferation, Apoptosis, JNk-2, NF-κB

## Abstract

**Objective:**

This study aims to investigate the *in vitro *effects of Ulinastatin (UTI) and Taxotere (TXT) on cell proliferation; cell apoptosis; xenografted tumor growth; and expression of insulin-like growth factor receptor 1 (IGF-1R), platelet-derived growth factor A (PDGFA), nerve growth factor (NGF), c-Jun N-terminal kinase 2 (JNk-2), and NF-κB in a human primary breast cancer cells and breast cancer cell line MDA-MB-231.

**Methods:**

The cell lines cultured were divided into four groups: 1) control group, 2) UTI group, 3) TXT group, and 4) UTI+TXT group. The method of MTT essay, flow cytometry, and RT-PCR were used to detect cell proliferation, cell apoptosis, and expression of IGF-1R, PDGFA, NGF, NF-κB, JNk-2, respectively. The growth of xenografted tumor in nude mice was used to calculate the anti-tumor rate. Immunohistochemistry staining (SP) was used to detect the expression of IGF-1R, PDGFA, NGF, ki-67, caspase-3, JNk-2, and NF-κB.

**Results:**

Proliferation of human breast cancer cells and MDA-MB-231 cell lines, and growth rate of xenografted tumor decreased in order of UTI+TXT > TXT > UTI > control, apoptosis increased in the order control < UTI < TXT < UTI+TXT. The gene expression and protein expression of IGF-1R, PDGFA, NGF, NF-κB and JNk-2 in breast cancer cells was inhibited by UTI and TXT.

**Conclusions:**

UTI 1) inhibits the proliferation of human breast cancer cells and the growth of xenografted tumors, 2) induces cancer cell apoptosis, and 3) enhances the anti-tumor effect of TXT. This mechanism might be related to decreasing signal transduction of JNk-2 and NF-κB, and then expression of IGF-1R, PDGFA, NGF.

## Introduction

Breast cancer is a major malignant tumor threatens women's health. It is the second leading cause to women's death [1]. Ulinastatin (UTI), a physiological urinary trypsin inhibitor, inhibits a variety of proteases. It is widely used in treatment of inflammatory diseases, including disseminated intravascular coagulation, shock, and pancreatitis [2,3]. Our previous study showed that UTI exerts significant inhibitory effects on 1) the proliferation and invasion of human breast cancer cell lines MCF-7 and MDA-MB-231, 2) the growth of MCF-7 transplanted tumor in nude mice, 3) the gene and protein expression of CXCR4 and MMP-9 in breast cancer cells; UTI also enhances the anti-tumor effect of the chemotherapy drug cyclophosphamide [4,5]. TXT is the most effective chemotherapy drug to treat breast cancer. It is widely used on the treatment of metastatic breast cancer. In addition, it is a novel adjuvant chemotherapy for breast cancer patients [6]. In this study, we detected the inhibitory mechanisms of UTI on breast carcinoma growth via observations in *in vivo and in vitro *experiment of effects of UTI and TXT on the expression of human breast cancer cell lines, xenografted tumor, and insulin-like growth factor receptor 1 (IGF-1R), platelet-derived growth factor A (PDGFA), nerve growth factor (NGF).

## 1. Materials and methods

### 1.1 Cell line and animals

Human breast cancer cell line MDA-MB-231 (ER-) was a generous gift from the Shanghai Institutes for Biological Sciences, Chinese Academy of Sciences. The total 95 female BALB/c nu/nu mice aged 4-6 weeks old and weighing 17-21 g were provided by Chongqing Medical University Animal Research Center (Production License No. SCXK [Beijing] 2005-0013; Usage Permission No. SYX [Chongqing] 2007-0001).

### 1.2 Major reagents and apparatus

UTI was a generous gift from Techpool Bio-Pharma (Guangzhou, China); TXT was a generous gift from Sanofi-Aventis. The RT-PCR kit was purchased from TAKARA. Anti-IGF-1R antibody and anti-PDGFA antibody were purchased from Bioworld Technology (USA). Anti-NGF antibody, anti-ki-67 antibody, anti-caspase-3 antibody, anti-JNk-2 (c-Jun N-terminal-kinase-2) antibody, and anti-NF-κB were purchased from Abcam Company. CA15-3 monoclonal antibody was purchased from Zhongshan Goldenbridge Biotechnology (Beijing, China). A Facsvantage SE flow cytometer was purchased from BD Company (USA); the Gel Doc XR quantity one gel image analyzer was purchased from Bio-Rad Company (Hercules, CA, USA) and the CX40 fluorescent invert microscope was purchased from Olympus (Tokyo, Japan).

### 1.3 Cell culture and nude mice breeding

A female breast cancer patient, aged 72 years, without chemotherapy and particular previous medical history, was treated by Breast & Thyroid & Pancreas Surgery in Second Affiliated Hospital of Chongqing Medical University. A specimen was taken from the patient's breast, which had undergone radical mastectomy. The pathology results revealed an infiltrating ductal carcinoma; immunohistochemistry revealed ER (+), PR(++), CerbB-2(-). The breast carcinoma specimen was sent to the lab within 2 h and cut into 1-mm^3 ^pieces. The sample was digested for 12 h in a mixture of 1% collagenase II plus hyaluronidase at 37°C, the supernatant was discarded, and the sample without supernatant was centrifuged at 1000 r/min for 5 min. A single breast carcinoma cells was collected, diluted to a concentration of 10^5^/mL, and then cultured in RPMI 1640 + 10% fetal bovine serum culture medium. Trypan blue stain was used to assess cell viability, and vivid breast carcinoma cells were taken to descendence. Cell adherence was used repeatedly to remove cell impurities [6]. Human breast cancer cell line MDA-MB-231 was cultured in RPMI-1640 medium plus 10% fetal bovine serum, 100 U/mL penicillin, and 100 mg/L streptomycin at 37°C in an incubator with 5% CO_2 _and saturated in a humidity environment. The cultured cells within logarithmic growth were used in this study. Cell suspensions were prepared by trypsin digestion. Nude mice were kept in a specific pathogen free environment with a temperature of 22-25°C and 50-65% humidity. Drinking water, feed, and experimental materials were disinfected by sterilization, and the rule of aseptic operation was strictly followed. Our research reported in the manuscript has been performed with the approval of Chongqing Medical University ethics committee.

### 1.4 Immunocytochemical fluorescent staining

For fluorescent staining, 1 × 10^5 ^cultured cells were planted onto cover glass. The cover glass was removed when the cells covered 80% of the glass. After being fixed, the cover glass was 1) used to hatch inactive endogenous enzyme, 2) treated in 0.1% Triton liquid, 3) washed within phosphate-buffered saline (PBS), 4) subjected to immunocytochemical and immunofluorescent staining according to instructions for CA15-3 primary antibody (1:100) and fluorescein isothiocyanate-marked secondary antibody (1:100), 5) sealed with glycerine, 6) inserted into an Olympus CX40 inverted microscope for observation and recording.

### 1.5 Grouping and drug administration

#### 1.5.1 Cell experiment

Cells were divided into four groups: 1) the control group was treated with physiological saline only; 2) the UTI group was treated with UTI at the concentration of 800 u/mL [5]; 3) the TXT group was treated with TXT at the concentration (same as the average peak plasma concentration) of 3.7 μg/mL [7]; and 4) the UTI+TXT group was treated with UTI and TXT at the same concentrations described above. All drugs were prepared 6 h before administration.

#### 1.5.2 Animal experiment

After being harvested, the cell lines washed with PBS and resuspended in serum-free RPMI-1640 medium. The cell concentration was adjusted to 1 × 10^7 ^cells/mL. Cells were inoculated subcutaneously into the right armpits of 45 nude mice at 0.2 mL/mouse. 21 days after inoculation, animals with tumor volumes ≥ 500 mm^3 ^were chosen in the study. A total of 28 animals were randomly divided into four groups for subsequent intraperitoneal injections as follows: 1) The UTI group (*n *= 7) was injected with UTI at 1600 U/day/mouse for 20 consecutive days [4]; 2) the TXT group (*n *= 7) was injected with TXT at 20 mg/kg on days 1, 7, and 14 [7]; 3) the UTI+TXT group (*n *= 7) was injected with UTI and TXT at dosages of UTI and TXT groups described in 1.5.1; and 4) the control group (*n *= 7) was injected with an equal volume of saline in 1.5.1 for 20 days. The animals were sacrificed for sample collection 21 days after administration. Minimum (D) and maximum (L) tumor diameters were measured to calculate the tumor volume (V), drawn the growth curve, and calculate the tumor inhibition rate. The *q *was also calculated via King's formula (*a *is the inhibition rate of UTI, *b *is the inhibition rate of TXT, and *c *is the inhibition rate of group UTI+TXT; *q *> 1.15 represents a synergistic effect, 1.15 >*q *> 0.85 represents an additive effect, and *q *< 0.85 represents an antagonistic effect). The related formulas are as follows:

1) tumor volume (cm^3^) = (L^2 ^× D)/2;

2) tumor inhibition rate (%) = [1 -(starting average tumor volume of treatment group - ending average tumor volume of treatment group)/(starting average tumor volume of control group - ending average tumor volume of control group)] × 100%;

3) *q *= *c*/ [(*a *+ *b*) - *a *× *b*]. 

After being harvested, MDA-MB-231 cells were washed twice with PBS, and then resuspended in serum-free RPMI-1640 medium. The cell concentration was adjusted to 2.5 × 10^10 ^cells/L. Cells were inoculated subcutaneously into the right armpits of 50 nude mice at 0.2 mL/mouse. The method was the same as the experiment described above.

### 1.6 Detection of cell proliferation by MTT

Cultured cells were inoculated into 96-well plate at 1.5 × 10^3 ^cells/well and divided into four groups as described in 1.5. Cells were cultured for 24, 48, or 72 h in a 37°C humid environment with 20 μL MTT solution (5 mg/mL). After another 4 h of culturing at 37°C, the culture medium was removed, 200 μL dimethyl sulfoxide was added to each well, and the plates were incubated for 10 min with shaking. The absorbance of each well was detected with an enzyme-linked immunosorbent assay microplate reader at a wavelength of 570-nm, and then the growth inhibition rate was calculated. All experiments were repeated 3 times under the same conditions.

### 1.7 Detection of cell apoptosis by flow cytometry

Cells were inoculated into a 25-mL flask and treated with drugs as described in 1.5 when they covered 80% of the flask. After being treated for 48 h, cells were digested by trypsin, collected by centrifuge, resuspended in an EP tube with PBS, and fixed in 1% polymerisatum. Before being used in the experiment, the cells were washed three times in PBS, added Annexin-V/PI stored in 4°C, stood at room temperature without light for 3 min, and were filtered in 300-mesh filter traps. Flow cytometry (Facsvantage SE; BD) was used to analyze cell apoptosis.

### 1.8 Reverse-transcribed quantitative PCR detection of IGF-1R, PDGFA, NGF, NF-κB, and JNK2 mRNA expression in primary breast cancer cells and breast cancer cell line MDA-MB-231

Cells were inoculated into four 75-mL flasks (5 × 10^5 ^cells/mL) and cultured for 48 h in RPMI-1640 culture medium plus 10% fetal bovine serum. After removing the original medium, cells were treated for 48 h with drugs as described in 1.5. Total RNA in all experimental groups was isolated with RNAiso Plus following instructions. The concentration and purity of isolated total RNA was measured by ultraviolet spectrophotometry. The cDNA was then reverse-transcribed according to the instructions in the reagent kit and amplified via PCR with β-actin and glyceraldehyde 3-phosphate dehydrogenase (GAPDH) as inner consults. Primer design software Primer 5.0 from Shanghai Biotechnology (Shanghai, China) was used to design the primer.

The primer sequence was as follows. Up primer of IGF-1R: 5'TGGAGTGCTGTATGCCTCTGTG-3', down primer of IGF-1R: 5'-GTGGACGAACTTATTGGCGTTG-3', amplified product: 493 bp. Up primer of PDGFA: 5'-CCCGCAGTCAGATCCACAGCAT-3', down primer of PDGFA: 5'-TTCCCGTGTCCTCTTCCCGATA-3', amplified product: 483 bp. Up primer of NGF: 5'-CCCCCTTCAACAGGACTCAC-3', down primer of NGF: 5'-GGTCTTATCCCCAACCCACA-3', amplified product: 110 bp. Up primer of NF-κB: 5'-CTTCAGAATGGCAGAAGATGA-3', down primer of NF-κB: 5'-CACATACATAACGGAAACGAAA-3', amplified product: 191 bp. Up primer of JNK2: 5'-TGCGTCACCCATACATCACT-3', down primer of JNK2: 5'-TGCTTCTTTCTTCCCAATCC-3', amplified product: 156 bp. Up primer of GAPDH: 5'-ATCAACGGGAAACCCATCAC-3', down primer of GAPDH: 5'-CGCCAGTAGACTCCACGACAT-3', amplified product: 98 bp. Up primer of β-actin: 5'-CACCCGCGAGTACAACCTTC-3', down primer of β-actin: 5'-CCCATACCCACCATCACACC-3', amplified product: 207 bp. The reaction conditions were as follows: denaturation at 94°C for 30 s, at 58°C for 30 s, and at 72°C for 1 min, for a total of 35 cycles. A total of 5 μL test factor and internal amplified product were separately subjected to agarose gel electrophoresis and analyzed via the Gel Doc-XR quantitative analysis system. Cell expression was represented as the ratio of amplified integrated gene absorption to internal gene absorption.

### 1.9 Detection of protein expression in IGF-1R and PDGFA via western blotting

Cell protein samples in each experimental group were collected by western cell lysate. Collected protein samples were 1) expanded by polyacrylamide gel electrophoresis; 2) blotted onto polyvinylidene fluoride membrane by electroporation; 3) hatched at room temperature for 2 h with anti-IGF-1R (1:500) antibody, anti-PDGFA (1:500) antibody, or membrane; 4) treated with horseradish peroxidase and enzyme-labeled secondary antibody; 5) subjected to color reaction via the enhanced chemiluminescence hypersensitive chemiluminescence method. The optical band concentration was analyzed and recorded with the Gel Analysis System. Detection of relative protein strength was represented in the ratio of the optical protein band concentration to the internal gene β-actin.

### 1.10 Detection of protein expression in xenografted tumor tissue in nude mice by immunohistochemistry (Staining Horseradish Proxidase)

Xenografted tumors from sacrificed nude mice were collected for immunohistochemical analysis. The appearance of brown granules in the cytoplasm was considered positive for protein. The integrated optical concentration of slides in each group was analyzed via Image-Pro Plus 6.0.

## 2. Statistical analysis

All data were analyzed with SPSS 18.0 and represented as $\bar{x}$ ± s. A completely randomly designed analysis of variance was used to compare the data among groups, and differences of *P *< 0.05 were considered statistically significant.

## 3. Results

### 3.1 Growth, morphology, and appraisal of breast carcinoma cells

**Figure 1:**
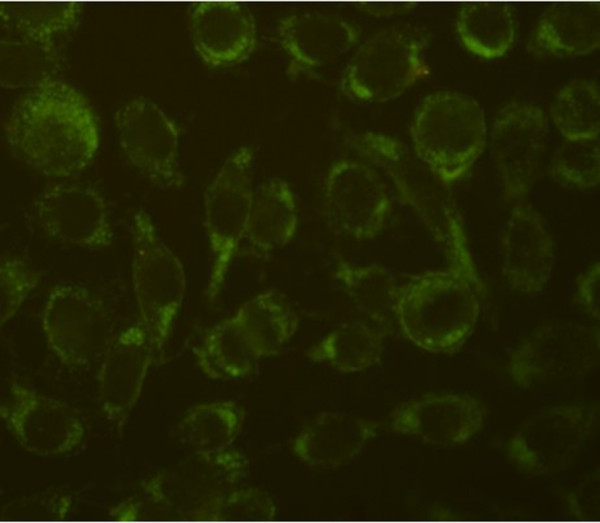
**Positive expression of primary cultured cell CA15-3 (400×)**.

The cultured breast carcinoma cells showed stable proliferation after 2 weeks by adhering to the wall in long shuttle shapes, while some interstitial cells showed in polygon stretching growth, sometimes the cell fragments and dross covered there. Differential adhesion was used to remove the interstitial cells and fibroblasts. Breast carcinoma cells were those whose cell viability reached 90% as detected by trypan blue stain and that achieved positive results for cytoplasmic glycoprotein in immunocytochemical staining (Figure 1).

### 3.2 Proliferation of breast carcinoma cells

**Figure 2:**
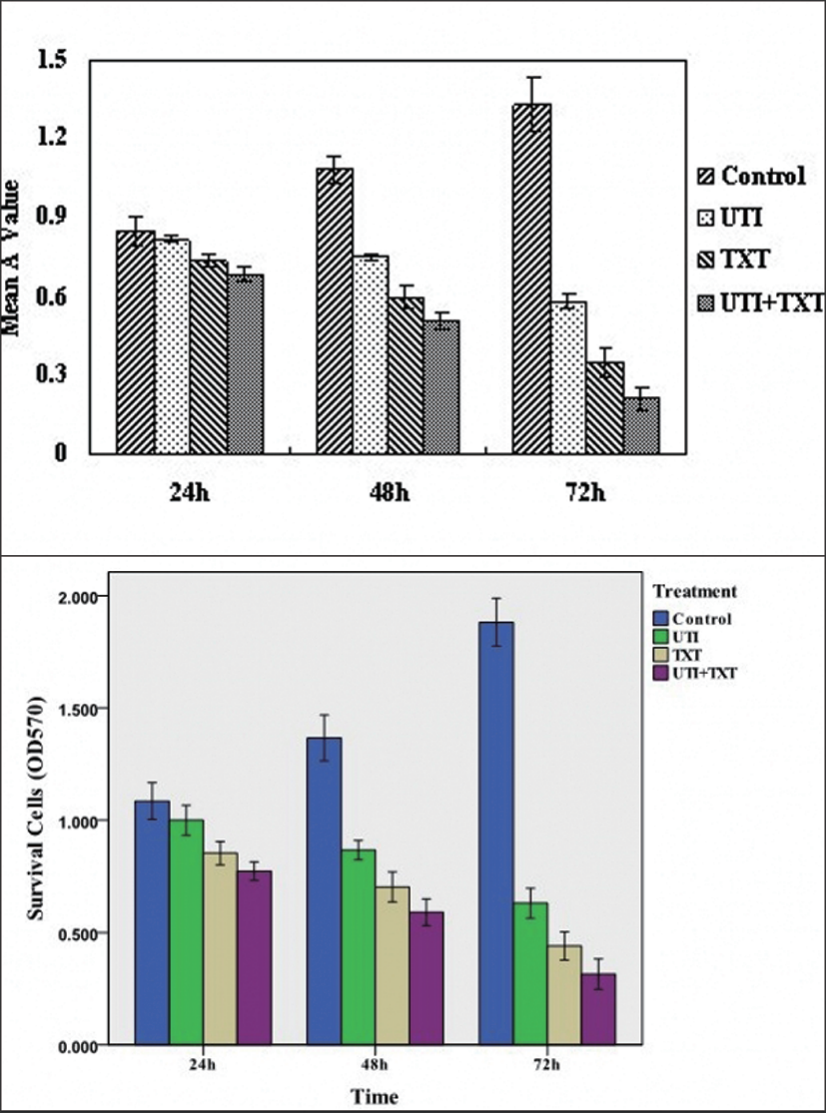
**(a). Effect of UTI and TXT on the proliferation of primary (ER+) breast carcinoma cells**. (b). Effect of UTI and TXT on the proliferation of MDA-MB-231 (ER-) breast carcinoma cells.

Primary breast carcinoma cells were treated with UTI, TXT, or UTI+TXT for 24-72 h, and the results showed that UTI, TXT, and UTI+TXT significantly inhibited the proliferation of breast carcinoma cells. These inhibitory effects were statistically significant compared with the control group (*P *< 0.05). In addition, the inhibitory effect was enhanced after extended treatment, which reveals a time-dependent effect (Figure 2a). UTI, TXT, and UTI+TXT also significantly inhibited the proliferation of MDA-MB-231 cells compared with the control group (*P *< 0.05), and the inhibitory effect was enhanced after extended treatment (*P *< 0.01). The strength of the inhibitory effects of the treatments was UTI+TXT > TXT > UTI. All differences were statistically significant (*P *< 0.01; Figure 2b).

### 3.3 Apoptosis rate of breast carcinoma cells

**Figure 3:**
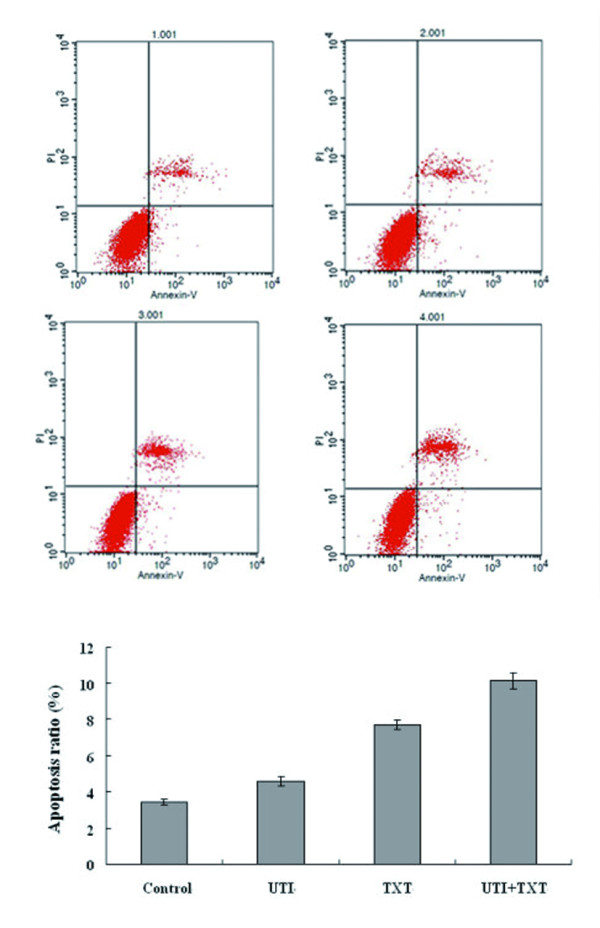
**Effect of UTI and TXT on the apoptosis rate of primary breast carcinoma cells**.

**Figure 4:**
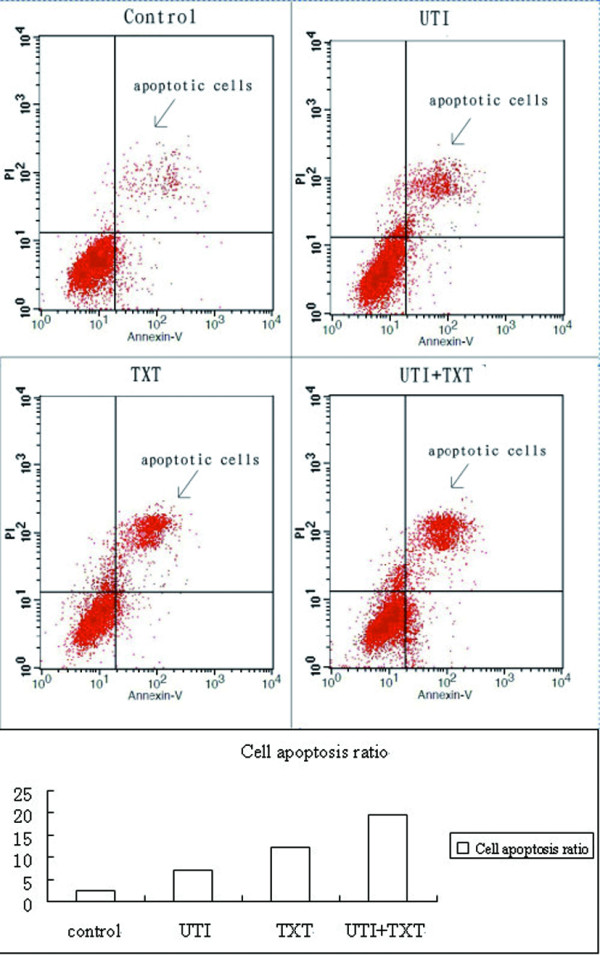
**Effect of UTI and TXT on the apoptosis rate of MDA-MB-231 breast carcinoma cells**.

After being treated with UTI, TXT, or UTI+TXT for 48 h, apoptosis rates of primary breast carcinoma cells were 4.562% ± 0.263, 7.683% ± 0.253, and 10.115% ± 0.123, respectively. Compared with the control group (3.426% ± 0.156), UTI, TXT, and UTI+TXT significantly induced the apoptosis of breast carcinoma cells (*P *< 0.05); the effect on UTI+TXT was strongest (Figure 3). UTI, TXT, and UTI+TXT also significantly induced the apoptosis of MDA-MB-231 breast carcinoma cells (*P *< 0.05), and effect on UTI+TXT was strongest (Figure 4).

### 3.4 Protein expression of IGF-1R and PDGFA in breast carcinoma cells

**Figure 5:**
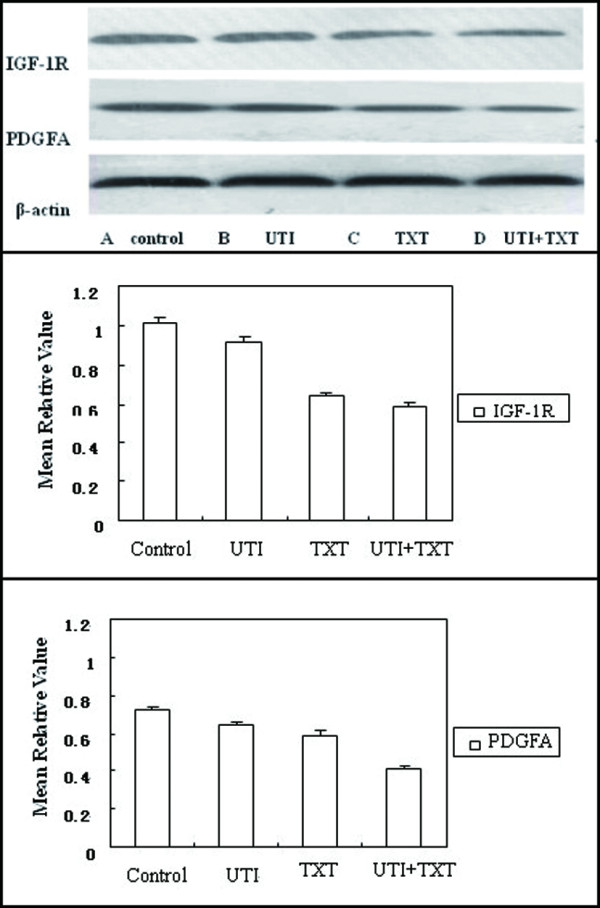
**Effect of UTI and TXT on protein expression levels of IGF-1R and PDGFA in primary breast carcinoma cells**.

Western blotting showed that after primary breast carcinoma cells were respectively treated with UTI, TXT, and UTI+TXT for 48 h, the protein expression of IGF-1R and PDGFA decreased significantly compared with the control group (*P *< 0.05; Figure 5) in the order of UTI+TXT > TXT > UTI. There are synergetic effects in UTI+TXT, either.

### 3.5 Gene expression of IGF-1R, PDGFA, NGF, NF-κB, and JNK2 in breast carcinoma cells

**Figure 6:**
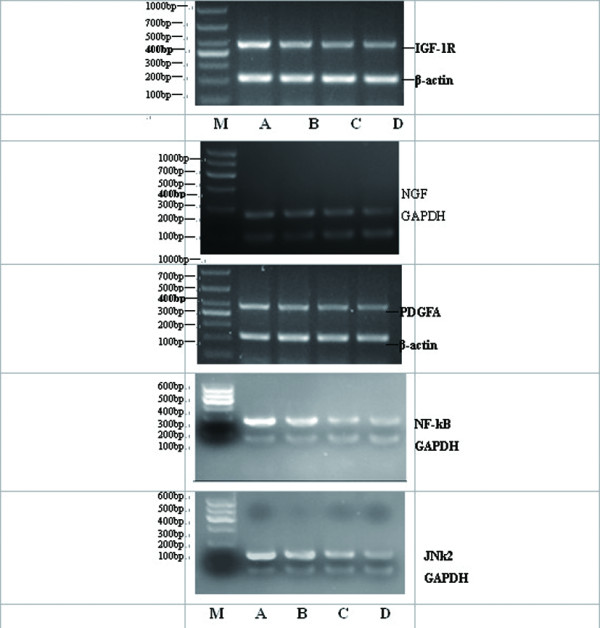
**Line of gene expression in IGF-1R/β-actin, NGF/GAPDH, PDGFA/β-actin, NF-kB/GAPDH, JNk2/GAPDH**. Note: M): DL1000 Marker; A): control group; B): UTI group; C): TXT group; D): UTI+TXT group.

**Figure 7:**
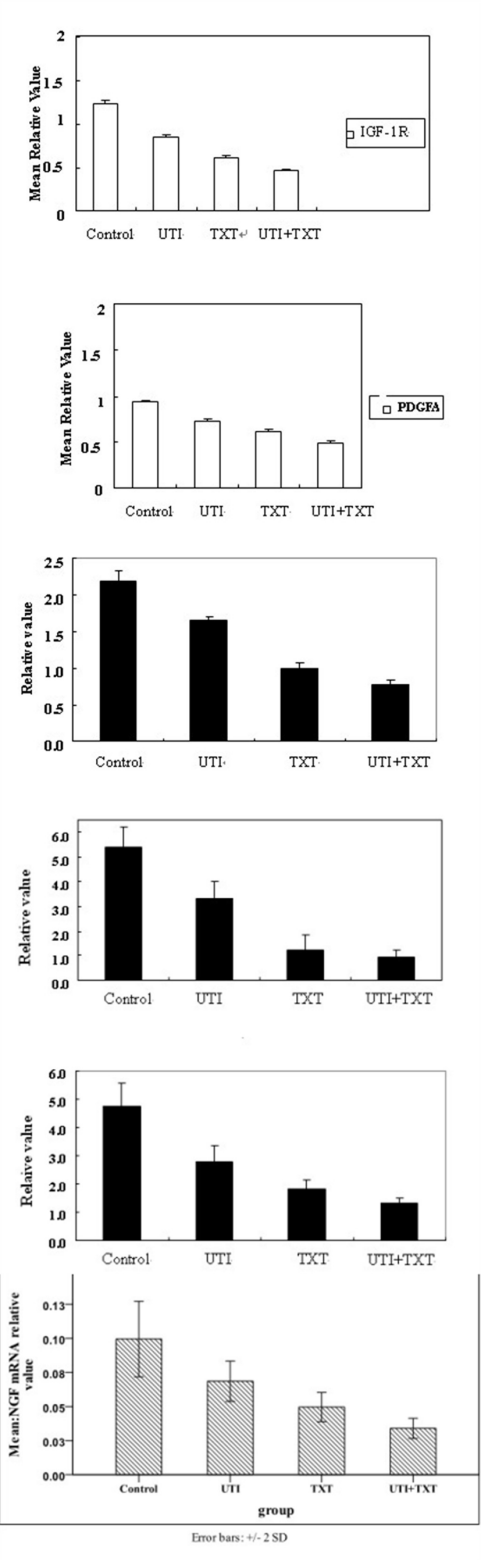
**(a). Gene expression of IGF-1R in primary breast carcinoma cells. (b). Gene expression of PDGFA in primary breast carcinoma cells**. (c). Effect of UTI and TXT on gene expression of NGF in primary breast carcinoma cells. (d). Effect of UTI and TXT on gene expression of NF-κB in primary breast carcinoma cells. (e). Effect of UTI and TXT on gene expression of JNk-2 in primary breast carcinoma cells. (f). Effect of UTI and TXT on gene expression of NGF in MDA-MB-231 breast carcinoma cells.

After being respectively treated with UTI, TXT and UTI+TXT for 48h, the gene expression of IGF-1R, PDGFA, NGF, NF-κB, and JNK2 in human breast cancer cells decreased significantly compared with the control group (*P *< 0.05; Figure 6, Figure 7a, b, c, d, e) in the order of UTI+TXT > TXT > UTI > control. UTI, TXT, and UTI+TXT also significantly inhibit the NGF mRNA expression on MDA-MB-231 breast carcinoma cells compared with the control group (*P *< 0.05). However, the difference in NGF mRNA expression between the TXT and UTI+TXT groups was not statistical significant (*P *= 0.055; Figure 7f).

### 3.6 Effects of UTI and TXT on the growth of xenografted breast tumor in nude mice

**Figure 8:**
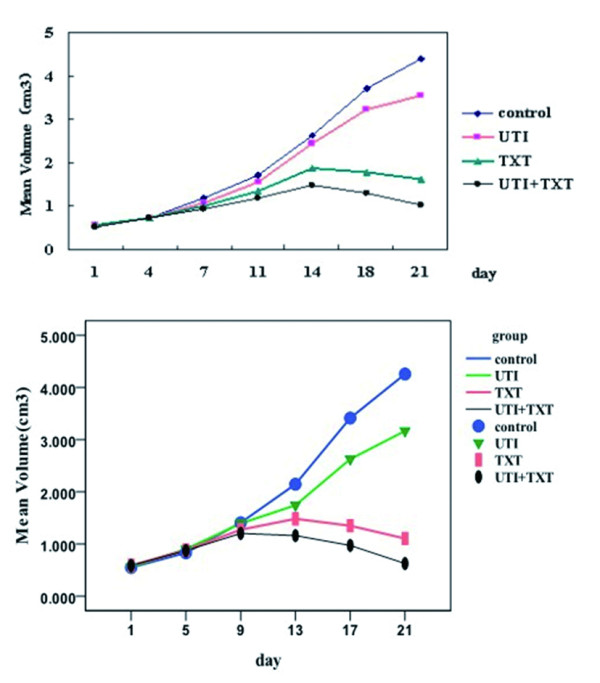
**(a). Growth curve for primary breast cancer cell xenografted tumors**. (b). Growth curve for MDA-MB-231 breast cancer cell xenografted tumors.

A total of 2 mice (1 in the control group and 1 in the UTI group) died after the drug treatment because of tumor-associated extreme consumption and cachexia. The growth curve of primary breast transplanted tumors showed that the average tumor volume of the mice in the control and UTI groups was not markedly reduced; however, UTI delays the increase in transplanted tumor volume (*P *< 0.05). In contrast, the average tumor volume in animals in the TXT and UTI+TXT groups gradually reduced over time after 11 d in the order of UTI+TXT > TXT (*P *< 0.05). King's formula was *q *= 1.088, implying an additive inhibitory effect of UTI and TXT on the growth of transplanted breast cancer in nude mice (Figure 8a). The growth curve of the MDA-MB-231 transplanted tumors was the same (Figure 8b).

### 3.7 Effects of UTI and TXT protein expression of PAFR, PDGFA, IGF-1R, NGF, NF-κB, and JNk-2 in xenografted tumors

**Figure 9:**
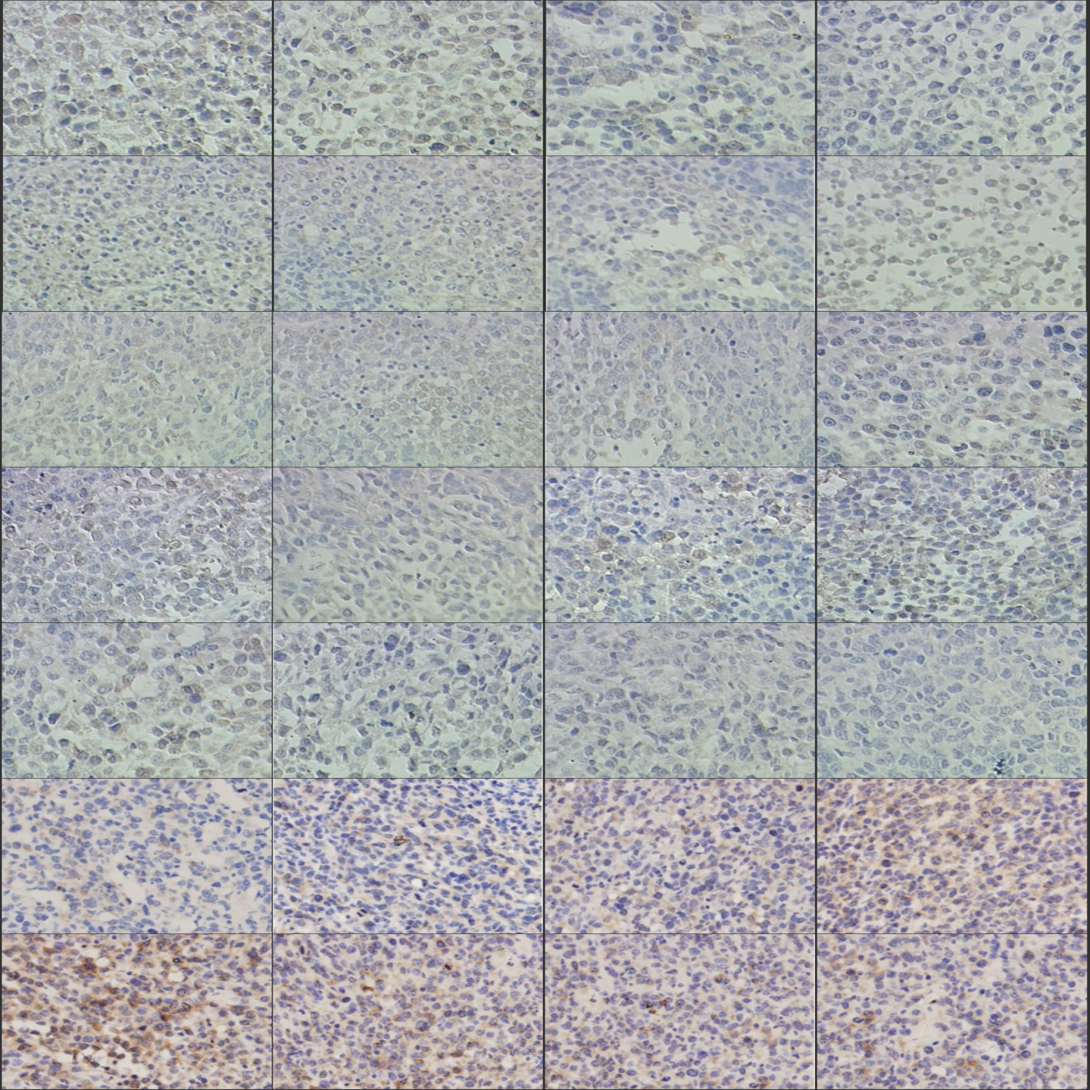
**Effect of UTI and TXT on the immunohistochemistry expression of IGF-1R, PDGFA, NGF, NF-κB, ki-67, caspase-3, JNk-2 in xenografted tumor tissue of nude mice**.

**Figure 10:**
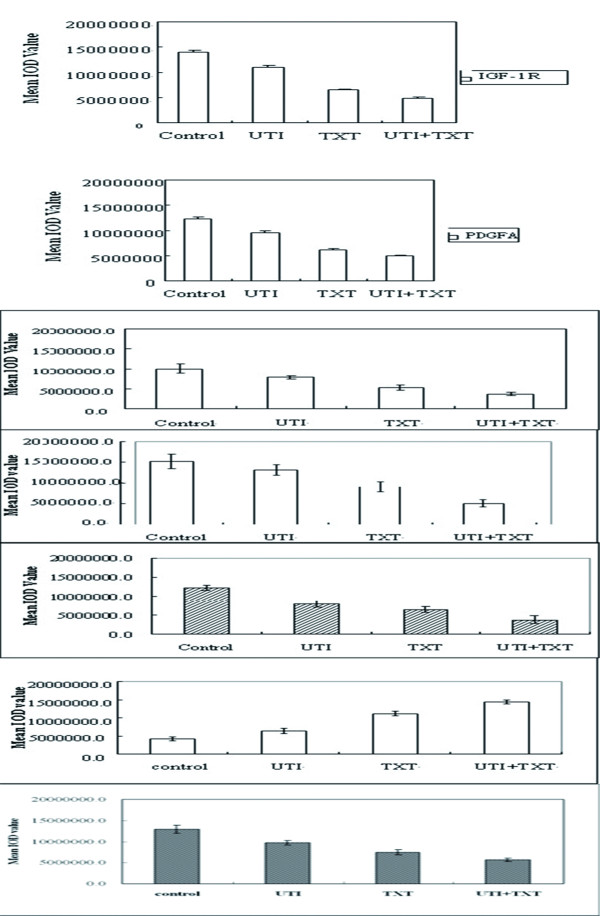
**Effect of UTI and TXT on the protein expression of IGF-1R, PDGFA, NGF, NF-κB, ki-67, caspase-3, JNk-2 in xenografted tumor tissue of nude mice**.

**Figure 11:**
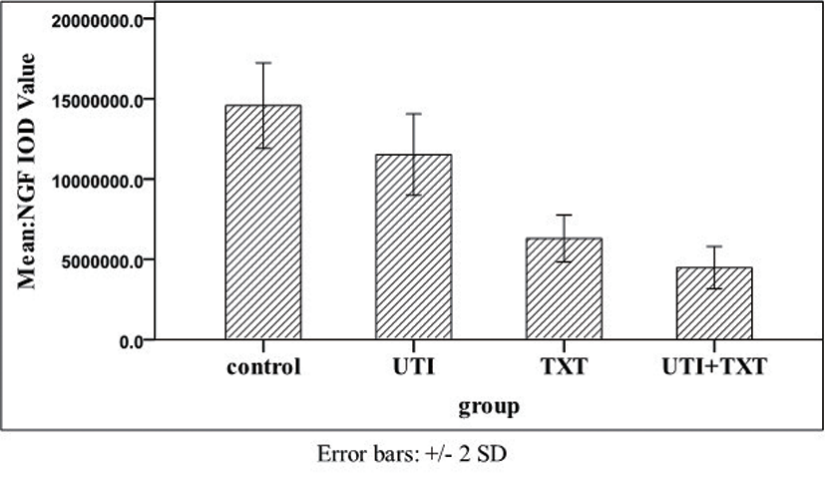
**Effect of UTI and TXT on the protein expression of NGF in MDA-MB-231 xenografted tumor tissue of nude mice**.

Immunohistochemistry showed that UTI, TXT, and UTI+TXT significantly inhibited the protein expression of PDGFA, NGF, and IGF-1R compared with the control group (*P *< 0.05). The inhibitory effect of UTI+TXT was strongest. The expression of ki-67, JNk-2, and NF-κB was reduced in the UTI, TXT, and UTI+TXT groups; however, the protein expression of caspase-3 increased significantly, and this effect was strongest for UTI+TXT (*P *< 0.05;Figure 9, 10, 11).

## 4. Discussion

Primary culture is the first culture after obtaining tissue from donor. The advantage of primary culture is that most of the cell still displays the biological characteristics of the *in vivo *cells. The result from Koechli [8] reported that an *in vitro *experimental result has good correlation with *in vivo *chemotherapeutical reactions (sensitivity = 90%, specificity = 86%). Hence, the primary culture method is suitable for investigating differences in the biological features of tumor cells. Proliferation inhibition and apoptosis are key factors in tumor treatment. In the present experiment, the proliferation of primary (ER+) and MDA-MB-231 (ER-) breast carcinoma cells are inhibited in a time-dependent manner. In addition, apoptosis of breast carcinoma cells increase. The anti-tumor effect of UTI+TXT was stronger than when UTI or TXT was used alone. Thus, UTI can enhance the anti-tumor effect of TXT. ki-67 antigen is a nuclear antigen related to cell proliferation; its function is related to chromosomes and cell karyokinesis [9]. ki-67 can reflect the proliferation viability of carcinoma cells because it is strongly related to the development, metastasis, and prognosis of malignant tumor [10]. Caspase-3 is the most important executor of apoptosis in the caspase family. Cell apoptosis can be inhibited by inhibiting the viability and functioning of caspase-3. Activated caspase-3 has a strong capacity to induce apoptosis of tumor cells; the increasing expression level suggests the cell apoptosis [11]. In this experiment, the decrease in ki-67 expression and increase in caspase-3 expression in xenografted tumor is further proof of the ability of these proteins to inhibit proliferation and increase apoptosis of tumor cells.

JNk is a member of the mitogen-activated protein kinase (MAPK) family. JNK2 gene is located on 5q35 and mainly mediates *in vitro *stimulation signals, such as virus, toxin, cytokine, and environmental stimulation signals [12].

IGF-1R is highly expressed in many kinds of tumors and closely related to tumor occurrence, development, and apoptosis. Overexpression of IGF-1R can promote the growth of breast carcinoma cells, and it might be related to induction of tumor apoptosis and stimulation of an immune reaction to remove residual carcinoma cells [13]. Upon being combined with corresponding ligands, IGF-1R inactivates the BAD protein, a member of the bcl family, by activating the PI3K/Akt or Ras/Raf-1/MAPK family to avoid apoptosis. Meanwhile, IGF-1R can activate NF-κB viability and induce cell proliferation [14,15].

PDGF is a group of peptide growth factors encoded by the primary cancer gene c-sis. When PDGF combines with corresponding acceptors (PDGFR), it can phosphorylate cell membrane protein and induce cell malignant transformation. PDGFA/PDGFR-α functions via autocrine and paracrine signals to stimulate interstitial hyperplasia and indirectly promote tumor growth; in addition, it can promote cell proliferation by strengthening the response of IGF-1 [16,17]. PDGF can improve PI3K activity, stimulate the phosphorylation of MAPK and AKT, increase degradation of extracellular proteins, upregulate MMP-2/9 expression, promote cell proliferation, and avoid apoptosis [18,19].

NGF is a pluripotent polypeptide growth factor, strong mitogen related to the proliferation, invasion, and vascularization of breast carcinoma cells [20,21]. Dolle et al. showed that breast carcinoma cells can produce and overexpress NGF [22]. Combined with acceptors in the breast carcinoma cell membrane, NGF can induce proliferation and inhibit apoptosis of breast carcinoma cells via a series of cascade reactions and signal transduction, then stimulate breast carcinoma cells to produce more NGF, forming a malignant autocrine loop. MCF-7, T47-D, BT-20, and MDA-MB-231 breast carcinoma cells secrete NGF and express NGFR; when NGF combines with TrkA, an intracellular signal is sent via p21ras by phosphorylation and the ras-MAPK signal pathway is stimulated to influence gene transcription, translation and mediate cell growth [23,24]. In the present experiment, we find that UTI and TXT inhibit gene and protein expression of IGF-1R, PDGFA, NGF, NF-κB, and JNk-2 in breast carcinoma cells and the effect of UTI+TXT is strongest.

In conclusion, this experiment demonstrates that UTI and TXT inhibit proliferation of breast cancer cells and growth of xenografted breast tumors, induce apoptosis of breast cancer cells. UTI and TXT down-regulate the expression of mRNA and protein of IGF-1R, PDGFA, NGF, NF-κB, and JNk-2 in breast cancer cells and xenografted breast tumors. The effect of UTI+TXT is strongest. This suggests that UTI and TXT have synergistic effects. The mechanism might be related to a decrease in the signal transduction of JNk-2 and NF-κB, and then the expression of IGF-1R, PDGFA, NGF.

## Abbreviations

GAPDH: glyceraldehyde 3-phosphate dehydrogenase; IGF-1R: insulin-like growth factor receptor 1; JNk-2: c-Jun N-terminal kinase 2; MAPK: mitogen-activated protein kinase; MTT: 3-(4,5-Dimethylthiazol-2-yl)-2,5-diphenyltetrazolium bromide; NF-κB: Nuclear Factor-κB; NGF: nerve growth factor; PAFR: platelet activating factor receptor; PBS: phosphate-buffered saline; PDGFA: platelet-derived growth factor A; PDGFR: platelet-derived growth factor receptor; RT-PCR: Reverse Transcription Polymerase Chain Reaction; TXT: Taxotere; UTI: Ulinastatin.

## Competing interests

The authors declare that they have no competing interests.

## Authors' contributions

HW cultured the cells and tested the cell proliferation and apoptosis with MTT assay, XS cultured the cells, did medical statistics, revised and submit this manuscripts, FG, BZ and YZ tested gene expression of the cells, ZS designed this experiment and wrote this manuscript. all authors read and approved the final manuscript.
